# Clinical Study Support by Long-Term Stability Studies of Alpha_1_-Proteinase Inhibitor and Urea in Relevant Biological Matrices

**DOI:** 10.3390/ph18040572

**Published:** 2025-04-14

**Authors:** Andrea Engelmaier, Martin Zimmermann, Harald A. Butterweck, Alfred Weber

**Affiliations:** 1 Pharmaceutical Science, Baxalta Innovations GmbH, Part of Takeda, 1220 Vienna, Austria; andrea.engelmaier@takeda.com; 2 Plasma Derived Therapies R&D, Baxalta Innovations GmbH, Part of Takeda, 1220 Vienna, Austria; martin.zimmermann@takeda.com (M.Z.); harald.butterweck@takeda.com (H.A.B.)

**Keywords:** α_1_-proteinase inhibitor, α_1_-antitrypsin, functional activity, immunological protein measurement, long-term analyte stability, clinical study

## Abstract

**Background/Objectives**: According to recent guidelines, including the guideline on bioanalytical method validation issued by the European Medicine Agency, the stability of clinical analytes should be known. We summarize human α_1_-proteinase inhibitor (A1PI) and urea stability data in relevant matrices, as these analytes are usually measured in clinical A1PI studies. Methods: Stability samples with appropriate A1PI concentrations were prepared in a citrated human reference plasma pool and a matrix mimicking bronchoalveolar lavage (BAL) solution. These samples were kept at −20 °C and −60 °C for up to 24 months. A1PI protein was measured with a nephelometric method and an enzyme-linked immunosorbent assay using paired commercially available polyclonal antibodies. A1PI elastase inhibitory activity was determined with an elastase complex formation immunosorbent assay that combines A1PI complex formation with a solid phase-immobilized elastase and immunological detection of the formed A1PI-elastase complex and urea in samples kept at −20 °C only with an enzymatic assay. **Results**: Overall, the stability criterion (100 ± 20%) was met for the analytes A1PI protein and A1PI activity at both temperatures during storage of BAL-mimicking and plasma samples for 15 and 24 months, respectively; urea was stable in both matrices at −20 °C for 18 months. Plasma samples showed smaller drops in concentration over storage time than BAL-mimicking samples. As expected, the reduction of A1PI elastase inhibitory activity was more pronounced than that of A1PI protein. Interestingly, the analyte concentration did not significantly influence the results in either of the sample matrices. **Conclusions**: The data confirmed the appropriate stability of the three analytes in the matrices of citrated plasma and BAL-mimicking samples for at least up to 15 months.

## 1. Introduction

Human α_1_-proteinase inhibitor (A1PI; also α_1_-antitrypsin [AAT]) is a single-chain 51-kDa glycoprotein with 394 amino acids and three complex type N-glycans attached to the asparagines 46, 83, and 247 [1,2]. Differently branched sialylated N-glycans and clipping of the N terminus result in a characteristic pattern of isoelectric focusing [3]. In addition, a C-terminal-clipped variant that has lost its C-terminal lysine after cleavage by basic carboxypeptidases has been described [4,5], completing a series of naturally occurring variants that share the amino acid sequence of the core protein. Structurally, the globular protein with the overall dimension of 6.7 × 3.2 nm [6] contains three β-sheets—A, B, and C—and nine α-helixes [7].

A mobile loop of 20 amino acids, termed the reactive center loop (RCL), presents the active site Met358-Ser359 [8], which tightly binds to the active region of neutrophil elastase or, less strongly, to other serine proteases including, among others, proteinase 3, cathepsin G, plasmin, kallikrein, thrombin, ADAM17, and some caspases. A1PI is often seen as the prototype member of the superfamily of serpins (serine proteinase inhibitors) [9,10]. Because of its energy-rich and, therefore, metastable conformation, the inhibitory mechanism has been compared to a molecular mousetrap [11]. Initially, the protease forms a tight non-covalent complex followed by a nucleophilic attack on the A1PI’s RCL carbonyl carbon of Met358 (P1) by the catalytic serine of the protease. The formation of an ester bond between the protease’s serine and the peptide carbonyl carbon of the serpin’s P1 amino acid is followed by separation of the cleaved ends of the loop, which is accompanied, as a special feature of all serpins, by a remarkable conformational rearrangement [12]: the protease-cleaved loop is inserted into the A-sheet. The attached protease is rapidly displaced to the other end of the molecule. The newly inserted strand acts as an anvil on which the protease is smashed so that the protease loses almost half of its structure, while A1PI reaches a stable conformation. This suicidal inhibitory mechanism critically relies on the short length of the cleaved reactive loop, limited in all serpins to not more than 17 amino acids (P17-Pl) [13]. The whole inhibitory process is designed to distort the protease by disrupting the network of hydrogen bonds that makes up the catalytic architecture of the protease’s active site and squeezing the active serine away from its catalytic site. The resulting covalent A1PI-protease complex is stable and rapidly cleared in vivo from the circulation by a serpin-enzyme complex receptor [14,15,16].

A1PI diffuses from plasma, where it circulates at levels ranging from 0.9 to 1.75 g/L (17–34 µM) with a half-life of three to five days [17], into the lung. It is responsible for more than 90% of the anti-elastase protection of the lower respiratory tract. A1P1 deficiency was first described by Laurell and Eriksson in 1963 [18], who associated it with the early occurrence of emphysema. A few years later, Sharp et al. [19] unraveled the until then unknown association between liver cirrhosis and antitrypsin deficiency, while Turino et al. provided evidence that lung emphysema is related to unopposed proteolytic activity of neutrophil elastase [20]. Further milestones in the history of alpha_1_-antitrypsin deficiency (AATD) were set by Owen et al. [21] and Jeppsson [22] in 1976, who identified the substitutions E264V and E342K in the AATD-linked A1PI variants Pi*S and Pi*Z, respectively. The Pi*Z variant is responsible for more than 96% of AATD cases, while more than 20 A1PI variants are associated with lower circulating A1PI levels [23]. Nonclinical research culminated in the first clinical study, in 1981. Five patients with Pi*ZZ were treated with a human plasma-derived A1PI preparation [24]. Although this pivotal study only showed biochemical efficacy because the replacement therapy increased A1PI serum and lung levels and significantly raised the elastase inhibitory potency of bronchoalveolar lavage (BAL) solution, it paved the way for the FDA’s approval of the first plasma-derived A1PI concentrate, Prolastin (Bayer), and implemented what is now called AATD augmentation therapy [25,26,27,28,29,30,31]. Advanced X-ray lung imaging techniques supported by computational models have since greatly increased the informative value of clinical studies in terms of augmentation therapy efficacy [32,33,34].

Currently, four plasma-derived A1PI concentrates (Prolastin C [Grifols], Aralast NP [Takeda], Zemaira [CSL Behring], and Glassia [Kamada, marketed by Takeda]) are approved by the FDA for AATD augmentation therapy. Patients are treated once a week by intravenous administration of 60 mg A1PI/kg based on body weight. So far, a therapy with recombinant A1PI has not been established, mainly due to the protein’s glycosylation, which is indispensable for an adequate circulatory half-life. Nevertheless, a recently completed phase I study of the recombinant AAT Fc fusion protein INBRX-101 in adults with AATD demonstrated a satisfactory safety profile and an ability to maintain protective functional A1PI serum levels for an extended period [35].

Independent of its serpin function, various anti-inflammatory effects have been attributed to A1PI [36,37,38]. To mention only a few, A1PI was shown to enhance the interleukin-10 (IL-10) expression in monocytes [39], to regulate the neutrophil chemotaxis by binding to IL-8 [40], and to inhibit the proinflammatory leukotriene B4 neutrophil signaling [41]. Furthermore, beneficial effects of A1PI are discussed for the human diseases type-1 diabetes [42], graft-versus-host disease [43], and acute myocardial infarction [44]. It is reasonable to assume that, as well as clinical studies required to refine AATD augmentation therapy, future studies will address these interesting topics.

According to recent guidelines, including the guideline on bioanalytical method validation issued by the European Medicine Agency (EMA) [45], the long-term stability of clinical analytes should be known in the biological matrix of interest. In the case of A1PI, this requirement seems to be more than reasonable given the singularity of the molecule, characterized by its high-energy, metastable state conformation. This makes A1PI prone to aggregation, accompanied by loss of functional elastase inhibitory activity. Therefore, we designed and carried out real-time stability studies lasting for up to 24 months. In these studies, we measured A1PI protein with two immunological methods and functional A1PI activity in the relevant biological matrices of citrated human plasma and in low protein-containing samples mimicking human BAL solution. Furthermore, the stability of urea was determined during storage at −20 °C for both matrices, since urea concentrations, determined in corresponding plasma and BAL samples, serve to determine the dilution factor of the original epithelial line fluid recovered by the BAL procedure.

## 2. Results

The results section first provides the data from the plasma stability study obtained using the nephelometric method for the measurement of A1PI protein. Next, the A1PI protein data from the plasma stability study obtained by the enzyme-linked immunosorbent assay (ELISA) testing study are shown, followed by the functional A1PI activities determined with the elastase complex formation immunosorbent assay (ECFISA). A1PI protein and functional activity data determined in the BAL solution-mimicking samples with ELISA and ECFISA, respectively, follow, and data describing the stability of urea in plasma and solutions with low protein content conclude this section.

### 2.1. A1PI Protein Stability in Citrated Human Plasma as Measured with the Nephelometric Assay

**Figure 1:**
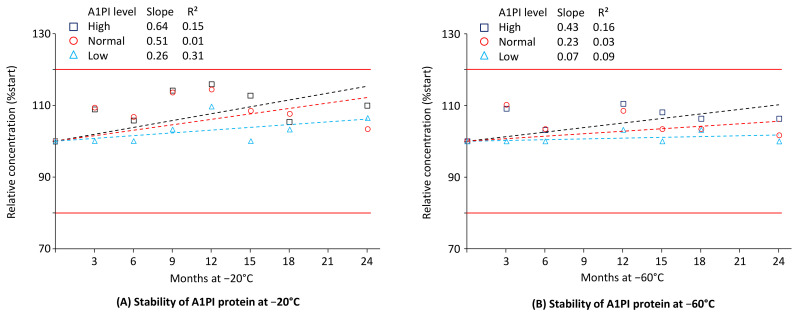
Stability of A1PI protein in the matrix of human citrated plasma, measured with the nephelometric method. The mean relative A1PI protein concentrations are expressed as a percentage of the respective starting concentrations (0.31 mg/mL, 1.18 mg/mL, and 2.86 mg/mL for the samples with low, normal, and high A1PI concentrations, respectively) and were determined at the individual time points by analysis of duplicates, which showed relative differences of less than 5%. Broken lines represent the regression lines calculated by linear regression analysis. Red lines mark the 100% ± 20% acceptance range, indicating stability as promoted by the EMA guideline on bioanalytical assay validation [45].

Nephelometric measurement of A1PI protein is highly suitable as a primary analysis method in clinical studies for several reasons. It possesses an adequate working range that effectively identifies A1PI deficiency states and delivers precise and accurate results in a significantly shorter time frame compared with ELISA. Both features encouraged us to measure A1PI nephelometrically in clinical samples with the Siemens Prospec BN instrument. We also used this method to measure three stability samples with high, normal, and low A1PI content in the matrix of human citrated plasma. Samples with high and with low A1PI content were prepared by adding the human purified A1PI preparation ARALAST NP or a human albumin preparation to a citrated reference plasma pool, respectively. Thus, the samples with the high A1PI level of approximately 3 mg/mL showed a three-times higher A1PI level than normal plasma. By contrast, the samples with the low A1PI level had A1PI concentrations of about 0.3 mg/mL, clearly below the cut-off level of 0.57 mg/mL used to identify A1PI-deficient patients [46]. Figure 1 shows the stability data for these three samples, kept at −20 °C and −60 °C over 24 months (see also Appendix A.5, Table A7).

A1PI protein, as measured with the nephelometric method, was shown to be stable in the matrix of human citrated plasma over the whole study period of 24 months when kept at −20 °C and −60 °C. During storage at each temperature, no individual data point suggested any loss in A1PI protein content. Thus, for the samples with normal and low A1PI levels kept at −20 °C, the recoveries of the starting A1PI protein levels were 103.4% and 106.5%, respectively, at the end of the study, while the samples with the high A1PI level showed a recovery of 109.8%. Similarly, the normal and low samples, kept at −60 °C, showed recoveries of 101.7% and 100.0%, respectively, at the end of the study. The samples with the high A1PI level demonstrated a recovery of 106.3%. These results most likely reflect the assay variability because the nephelometric method was shown to have a mean inter-assay precision of 4.3%. The regression lines calculated between relative A1PI protein concentrations and time had slightly positive slopes, but the low coefficients of determination show their high levels of uncertainty. Overall, the three citrated plasma samples, including one with an A1PI concentration indicative of A1PI deficiency, demonstrated stability during storage at −20 °C and −60 °C over a period of two years.

### 2.2. A1PI Protein Stability in Citrated Human Plasma as Measured with the ELISA

**Figure 2:**
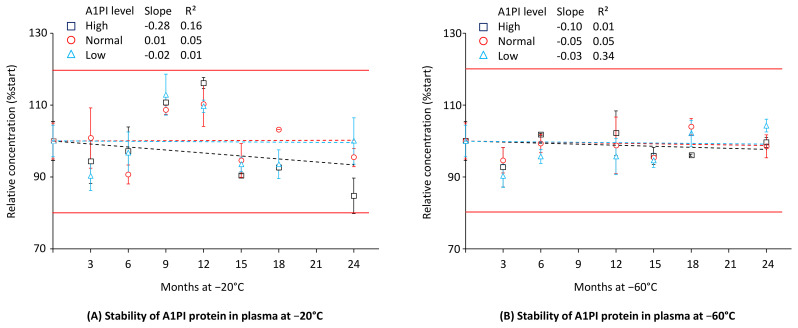
Stability of A1PI protein in the matrix of human citrated plasma, measured with the ELISA. The mean relative A1PI protein concentrations are expressed as a percentage of the respective starting concentrations (0.31 mg/mL, 1.28 mg/mL, and 3.54 mg/mL for the samples with low, normal, and high A1PI concentrations, respectively) and were determined at the individual time points by analysis of triplicates. Error bars mark the standard deviation (SD) of the means. Broken lines represent the regression lines calculated by linear regression analysis. Red lines mark the 100% ± 20% acceptance range, indicating stability as promoted by the EMA guideline on bioanalytical assay validation [45].

In contrast to the nephelometric method, which relies on the binding of only one antibody to the analyte A1PI, the enzyme-linked immunoassay (ELISA) uses two different antibodies. This at least doubles the number of possible binding interactions. The polyclonal antibodies used for the A1PI ELISA were developed in different species, increasing the probability that the ELISA might detect A1PI protein losses caused by the higher number of interactions required to obtain a signal. Therefore, we also measured the citrated human plasma A1PI stability samples with ELISA, even though the high sample dilutions, which had to be applied, could negatively affect the assay’s precision. Figure 2 shows the stability data for the three citrated human plasma samples with normal (~1 mg/mL), low (~0.3 mg/mL), and high (~3 mg/mL) A1PI levels, kept at −20 °C and −60 °C over 24 months (see also Appendix A.6, Table A8).

A1PI protein, as measured with the ELISA method, was shown to be stable in the matrix of human citrated plasma over the whole study period of 24 months when kept at −20 °C and −60 °C. During storage at each temperature, only two samples showed relative A1PI concentrations differing more than ± 15% from their initial concentrations. Thus, the samples with high A1PI levels, kept at −20 °C for 12 and 24 months, had relative A1PI concentrations of 116.1% and 84.7%, respectively. Given that the inter-run precision of the A1PI ELISA is 8.1%, it is plausible that these data, which pertain exclusively to the samples with the highest A1PI levels, requiring the highest dilutions for their analysis, could be attributed to assay variability. At the end of the study with −20 °C, the samples with normal, low, and high A1PI levels had relative A1PI concentrations of 95.4%, 100.0%, and 84.7%, respectively, while the corresponding relative A1PI levels were 98.5%, 104.3%, and 99.6%, respectively, at −60 °C. The regression lines showed slopes close to zero except for the samples with high A1PI levels kept at −20 °C. However, the low coefficients of determination show their high levels of uncertainty. Overall, the three citrated plasma samples, including one with an A1PI level indicative of A1PI deficiency, showed stability during storage at −20 °C and −60 °C over a period of two years.

### 2.3. A1PI Functional Activity in Citrated Human Plasma as Measured with the ECFISA

**Figure 3:**
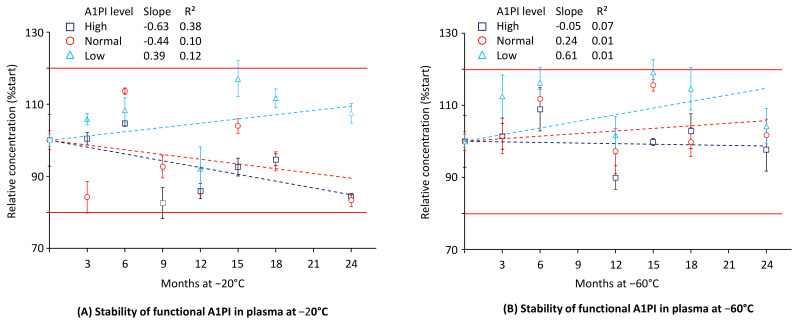
Stability of functional A1PI activity in the matrix of human citrated plasma. The mean relative functional A1PI activities are expressed as a percentage of the respective starting concentrations (0.24 mg/mL, 1.03 mg/mL, and 2.89 mg/mL for the samples with low, normal, and high A1PI concentrations, respectively) and were determined at the individual time points by analysis of triplicates. Error bars show the SDs of the means. Broken lines represent the regression lines calculated by linear regression analysis. Red lines indicate the 100% ± 20% acceptance range, indicating stability as promoted by the EMA guideline on bioanalytical assay validation [45].

Functional A1PI activity was determined by ECFISA. Figure 3 shows the data obtained for the three citrated human plasma samples with normal (~1 mg/mL), low (~0.3 mg/mL), and high (~3 mg/mL) A1PI levels, kept at −20 °C and −60 °C over 24 months (see also Appendix A.7, Table A9).

At each temperature, A1PI demonstrated stable functional activity over the whole period of 24 months, as all the relative concentrations determined differed by less than 20% from those initially determined. At the end of the study with −20 °C, the samples with normal, low, and high A1PI levels had relative A1PI concentrations of 83.4%, 107.5%, and 84.4%, respectively. The corresponding relative A1PI levels obtained for the samples kept at −60 °C were 101.7%, 104.2%, and 97.8%, respectively. Thus, storage of the citrated plasma samples at −60 °C resulted in about 10% lower functional activity losses than those observed at the higher temperature, but the assay variability challenges the significance of this finding. Interestingly, the lower A1PI concentration demonstrated a lower activity loss at −20 °C than the two other concentrations tested. This could also be related to the assay variable and not reflect real differences in stability. At the −20 °C storage conditions, the regression lines for the normal and high A1PI levels showed negative slopes of −0.44 and −0.63, indicative of progressing activity loss under these conditions. Based on these regression curves, the samples with normal and high A1PI levels would show 80% of their initial functional activity after 46 and 32 months, respectively.

### 2.4. Stability of A1PI Protein in BAL Mock Samples as Measured with the ELISA

**Figure 4:**
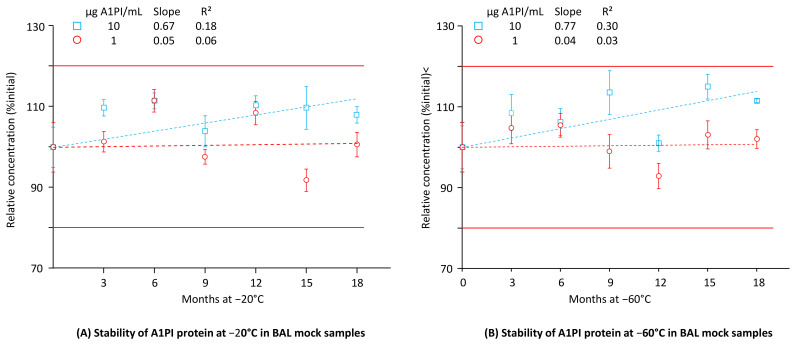
Stability of A1PI protein in the BAL mock samples. The mean relative A1PI protein concentrations, determined by triplicate analysis, are expressed as a percentage of the respective starting concentrations. Error bars mark the SDs of the means. Broken lines represent the regression lines calculated by linear regression analysis. Red lines mark the 100% ± 20% acceptance range, indicating stability as promoted by the EMA guideline on bioanalytical assay validation [45].

Low total protein concentration is a primary characteristic of BAL samples. Our BAL mock samples had a total protein concentration of about 50 µg/mL and thus displayed this feature at the low A1PI levels of 1 and 10 µg/mL. Figure 4 shows the A1PI protein data, obtained by the ELISA of the two BAL mock samples, kept at −20 °C and −60 °C over 18 months (see also Appendix A.8, Table A10).

A1PI protein, present at the low levels of 1 and 10 µg/mL, was shown to be stable in a sample matrix with a low total protein concentration at both −20 °C and −60 °C for 18 months. Thus, at the end of the study with −20 °C, relative A1PI protein concentrations of 100.7% and 108.0% were found for the 1 and 10 µg/mL samples, respectively. These samples, kept at −60 °C, showed relative A1PI protein concentrations of 102.0% and 111.5%, respectively. Interestingly, at both temperatures, the regression lines calculated for the samples with the lower A1PI concentration had slopes close to zero, indicating no substantial change in concentration over time. By contrast, those calculated for the higher A1PI concentration had positive slopes.

### 2.5. A1PI Functional Activity Measurement with the ECFISA in BAL Mock Samples

**Figure 5:**
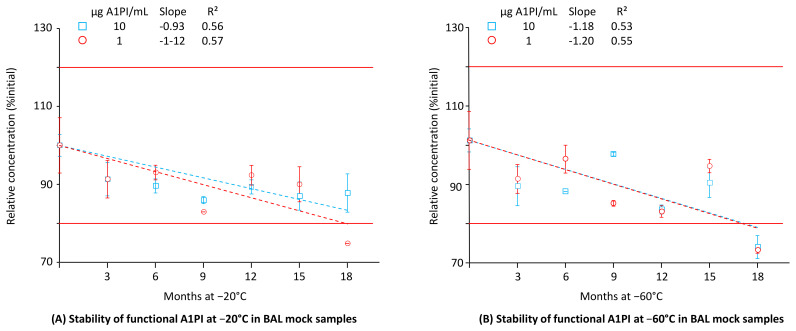
Stability of functional A1PI activity in BAL mock samples. The mean relative A1PI functional activities are expressed as a percentage of the respective starting concentrations and were determined at the individual time points by triplicate analysis. Error bars mark the SDs of the means. Broken lines represent the regression lines calculated by linear regression analysis. Red lines mark the 100% ± 20% acceptance range, indicating stability as promoted by the EMA guideline on bioanalytical assay validation [45].

Until now, the low A1PI concentration of BAL samples had hampered direct functional activity measurement, and before their analysis, such samples had to be concentrated. Direct measurement has become feasible owing to the increased sensitivity of the ECFISA. Figure 5 shows the functional A1PI activity data obtained by the ECFISA of the two BAL mock samples kept at −20 °C and −60 °C over 18 months (see also Appendix A.9, Table A11).

At both the A1PI concentrations and temperatures investigated, the functional A1PI activity of the BAL mock samples gradually decreased over time. Thus, after an 18-month storage period at −20 °C, the relative functional A1PI activities of the samples with 1 and 10 µg/mL were 74.7% and 87.8% of the initial ones, respectively. Storage at −60 °C did not significantly preserve the functional activity: the samples with 1 and 10 µg/mL showed 73.1% and 73.9% of their initial activity, respectively. The regression curves calculated between activity and time had obviously negative slopes ranging from −0.93 to −1.20, with coefficients of determination higher than 0.53. When kept at −60 °C, no apparent difference in stability was detected for the two concentration levels. At −20 °C, however, the higher A1PI concentration seemed to be more stable. Overall, the data demonstrated the stability of functional A1PI activity in a matrix with low protein content over 15 months.

### 2.6. Stability of Urea in BAL and Human Citrated Plasma

**Figure 6:**
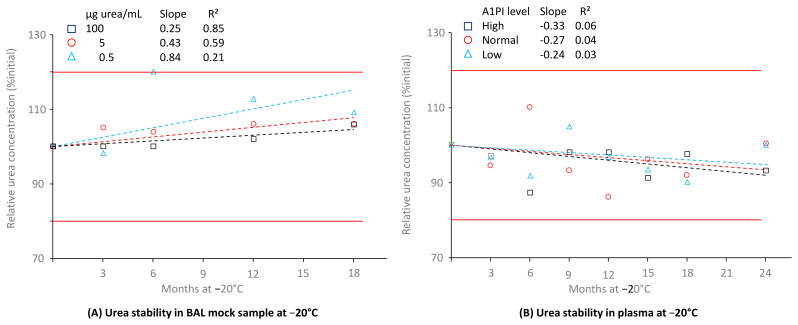
Stability of urea in BAL mock samples and citrated human plasma. The mean relative urea concentrations are expressed as a percentage of the respective starting concentrations and were determined at the individual time points by analysis of duplicates, which showed relative differences of less than 5%. Broken lines represent the regression lines calculated by linear regression analysis. Red lines mark the 100% ± 20% acceptance range, indicating stability as promoted by the EMA guideline on bioanalytical assay validation [45].

Determination of the urea concentration in BAL and corresponding plasma samples allows determination of the epithelial lining fluid dilution factor caused by the procedure to obtain BAL samples since urea can freely diffuse from plasma to the epithelial lining fluid. This factor is required to establish the transfer efficacy of A1PI to the lungs. Therefore, urea is measured. Figure 6 shows the stability of urea in BAL mock samples with a low protein level and in citrated human plasma samples, kept at −20 °C over 18 and 24 months, respectively (see also Appendix A.10, Table A12 and Table A13).

Urea was shown to be stable for 18 months at −20 °C in the samples with a low protein content, mimicking BAL samples, even at the low concentration of 0.5 µg/mL. There was no urea concentration-related difference in stability: the relative urea concentrations, expressed as a percentage of the initial concentrations, were 109.1%, 106.1%, and 105.9%, respectively, for the samples containing 0.5, 5, and 100 µg urea/mL. Similarly, urea was also stable in the citrated plasma matrix because the samples with low, normal, and high urea concentrations showed 100.0%, 100.4%, and 93.2%, respectively, of their initial urea concentrations after a 24-month period at −20 °C.

## 3. Discussion

Deficiency in the prototype serpin α_1_-proteinase inhibitor, detected by a paper electrophoretic serum pattern that almost completely lacked the α_1_-globulin band, was linked with the early development of lung emphysema and liver disease. This finding resulted in the establishment of a replacement therapy based on the weekly administration of 60 mg/kg A1PI, purified from human plasma. The association between a lack of A1PI and the development of disease seems to have stimulated research on this protein. The complete sequence of the cDNA [47] was published soon after the pivotal paper by Carell et al. [1], which discussed the structure and variation of human α_1_-antitrypsin. Just a few years later, the reason for the aggregate formation of the A1PI Z variant was identified as a so-called loop-sheet polymerization that involves the insertion of the reactive center of one molecule into the β-sheet A of another molecule. Polymerization of the A1PI Z variant occurs spontaneously in vivo at 37 °C and is responsible for low circulatory A1PI levels [48]. Due to the specific folding of the serpin superfamily proteins, which is characterized by a dominant β-sheet A and a mobile reactive center loop, normal M type A1PI is also prone to aggregation or the formation of latent forms. Both aggregated and latent forms are characterized by reduced functional activity [49]. The presence of such altered forms has also been described in a commercial A1PI product [50]. On the one hand, the energy-rich, metastable conformation, which is created by the specific folding and is shared by all serpins, provides the basis for the inhibitory suicidal mechanism of A1PI. On the other hand, it may be the reason for the loss of function inhibitory activity associated specifically with serpins. Other common mechanisms that are known to cause structural alterations of proteins and consequently induce loss of functional activity may include proteolytic cleavages and oxidation processes. As far as A1PI is concerned, the active site, though showing the highest affinity to neutrophil elastase, provides a broad inhibitory spectrum. Thus, not only other neutrophil proteases, including proteinase 3 and cathepsin G, but also the serin proteases plasmin, kallikrein, and thrombin and the intracellular cysteine proteases caspase-1, -3, and -6, are inhibited [40,51,52,53]. Because of the vast structural changes that accompany the A1PI-protease complex formation, A1PI not only loses its functional inhibitory activity, but antigenic epitopes are also altered or newly created. Thus, the reactivity with antibodies might be altered, which could be the reason for modified responses in immunological assays. Nevertheless, proteolytic cleavage by metalloproteases, released by activated neutrophils, has been described to inactivate A1PI. This cleavage occurs at position P7-P6 (Phe-Leu), close to A1PI’s reactive center, and creates cleaved A1PI with a loss of its functional activity. Similarly, oxidation of the methionine residues Met351 and/or Met358 in the reactive center loop has been shown to inactivate A1PI [8].

The specific features of A1PI highlighted above make stability studies for clinical samples appear indispensable, quite apart from guidelines that request stability studies for analytes determined in clinical studies. These studies become even more important when the two most prominent sample types of clinical A1PI trials are considered: citrated plasma and BAL. While analyses of citrated plasma samples, despite their complex protein composition, are not usually insurmountable tasks, the low total protein and low analyte concentrations, both characteristics for BAL samples, can turn such samples into a bioanalytical challenge. In the past, this challenge was resolved by subjecting such dilute samples to a concentrating step, which was carried out before analysis because the functional A1PI activity assay available required concentrations of at least 10 µg/mL. Regrettably, quantitative and qualitative protein losses have been reported for this procedure [54]. With the ECFISA used here, A1PI concentrations of as low as 1 µg/mL could be measured with acceptable accuracy and precision.

The most abundant proteins in BAL solutions are the proteins albumin (about 50% of total protein), transferrin (5 to 6%), α_1_-antitrypsin (3 to 5%), and the immunoglobulins G and A (together 30%), together making up almost 90% of the low total protein content. Thus, total protein levels of less than 100 µg/mL were found for human BAL samples, but their concentration mainly depends on the BAL procedure. These proteins mainly diffuse across the air–blood barrier from serum [55,56]. Minor protein components may be produced by different lung-resident cell types, including pulmonary T-cells, alveolar macrophages, bronchial epithelial cells, and others. Phospholipids represent the main components of surfactants, responsible for the decrease of alveolar surface tensions. Representative two-dimensional isoelectric focusing methods provided more than 1200 protein spots when visualized by silver staining. Yet, 93 proteins have been identified in human BAL solutions. A1PI usually accounts for 3% of the total protein present in BAL [57], with mean A1PI levels of 1.84 and 3.06 µg/mL reported for nonsmokers (*n* = 10) and smokers (*n* = 11), respectively [58].

Because no human BAL samples were available for our stability study, we decided to prepare and use BAL-mimicking surrogate samples by diluting a purified human A1PI preparation with bovine serum albumin. With a total protein concentration of 50 µg/mL at A1PI levels of 1 and 10 µg/mL present in saline, these samples appropriately mimicked the total protein and the A1PI levels of human BAL samples and their high salt concentration. The stability samples for the plasma study were obtained from a commercially available citrated human reference plasma pool. A1PI serum levels defining α_1_-antitrypsin deficiency with lung disease are usually lower than 0.57 mg/mL [46]. Therefore, the normal reference plasma pool was diluted with a purified human serum albumin so that samples with a reduced A1PI concentration of about 0.3 mg/mL were obtained to mimic the plasma samples obtained from an A1PI-deficient patient. Citrated human plasma samples with a high A1PI level of 3 mg/mL were obtained by adding a human purified A1PI preparation. Finally, urea stability samples (0.5, 5, and 100 µg/mL) were prepared in saline containing human serum albumin at a concentration of 50 µg/mL.

The data obtained in the stability studies demonstrated adequate and good long-term stability of A1PI protein and functional A1PI activity in the two sample matrices investigated during storage at −20 °C and −60 °C over at least 15 months, while urea was shown to be stable in both matrices during storage at −20 °C over 18 months.

Interestingly, the A1PI protein concentrations determined in citrated plasma with the nephelometric and the ELISA methods seemed to differ substantially, with the ELISA method providing lower concentrations. Thus, at the end of the study, the mean relative A1PI concentrations determined with the ELISA method were 93.4% and 100.8% at −20 °C and −60 °C, respectively, for the three A1PI levels, while 106.5% and 102.7% were determined with the nephelometric method. There was no significant difference, as shown by p-values of 0.08 and 0.51, for the means determined at −20 °C and −60 °C, respectively. The lower A1PI concentrations determined with ELISA could most probably be explained by the different test principles of both methods. While the signal in the nephelometric assay is the result of just one binding reaction, the ELISA used here requires two independent binding reactions to generate a response. Clearly, this feature of the ELISA enhances the sensitivity for detecting molecular alterations during long-term storage, as more epitopes of the analyte are involved.

Nevertheless, no pronounced stability difference was detected in either of the matrices for all three analytes at both temperatures. However, the three analytes—A1PI protein, API activity, and urea—showed different stability profiles, especially in the samples with the low protein and analyte concentrations mimicking BAL samples.

As expected, the functional A1PI activity progressively dropped at similar rates for both the A1PI levels and temperatures investigated, while the A1PI protein levels, measured with ELISA, remained stable. This clear loss of functional A1PI activity suggests that molecular alterations of A1PI were in play rather than surface adsorption phenomena, which can be reasonably excluded because of the stable A1PI protein levels. These alterations seemed to affect the serpin’s metastable “mousetrap” conformation required for effective complex formation with proteases. Since the citrated plasma samples did not demonstrate similar losses of functional A1PI activity over time, it seems reasonable to assume that the low total protein and the high salt concentration of the samples were responsible for this finding. It might, therefore, be worth considering adding a stabilizing inert protein or other stabilizing agents, such as polyethylene glycol or detergents, to BAL samples before their long-term storage.

In summary, the stability studies carried out for the three analytes—A1PI protein measured with two immunological methods, functional A1PI activity, and urea—provided evidence for their long-term stability in relevant clinical matrices.

## 4. Materials and Methods

### 4.1. Materials

The following chemicals from VWR (Vienna, Austria) were used: NaHCO_3_, Na_2_CO_3_, KCl, NaCl, KH_2_PO_4_, Na_2_HPO_4_ × 2 H_2_O, H_2_SO_4_ (95–97%), urea, water (HPLC grade), and HCl (25%). Tween 20 (EIA grade) was obtained from Bio-Rad (Vienna, Austria), bovine serum albumin (BSA, A0281) and benzamidine hydrochloride monohydrate (B6506) from Sigma (Vienna, Austria), non-fat dry milk from Maresi (Vienna, Austria), Patentblau V from Chroma-Waldeck (Münster, Germany), and the tetramethylbenzidine peroxidase substrate SureBlue from KPL (Medac; Hamburg, Germany). The biological reagents and standards used are described with the respective methods.

### 4.2. Preparation of Stability Samples and Design of the Stability Study

The stability of A1PI was investigated in a human citrated plasma pool at subnormal (~0.3 mg/mL), normal (~1 mg/mL), and elevated (~3 mg/mL) A1PI levels. For the stability samples with a subnormal A1PI level, the reconstituted lyophilized reference plasma pool 1R31 (Technoclone, Vienna, Austria) was diluted 1 + 2 with a purified 5% human serum albumin preparation (Takeda, Vienna, Austria; #0100301A). The purified A1PI preparation Aralast NP (Takeda, Vienna; #VNB5K036) was used to obtain stability samples with an elevated A1PI level. Aliquots of 200 µL were prepared and stored at −20 °C and −60 °C. A1PI protein (nephelometric and ELISA test), A1PI functional activity, and urea measurements were carried out every three months over two years.

Two BAL surrogate samples were prepared by diluting a purified human A1PI preparation (ARALAST NP VNB5J013; Takeda, Vienna, Austria) with bovine serum albumin in saline to achieve functional activity levels of 1 and 10 µg/mL at a total protein concentration of 50 µg/mL. Immediately after preparation, aliquots of 500 µL were filled in capped polypropylene tubes and stored at −20 °C and −60 °C. A1PI protein and functional activity were measured every three months over 18 months. The urea stability samples contained 0.5, 5, and 100 µg/mL in saline containing 50 µg human serum albumin/mL.

### 4.3. Nephelometric A1PI Protein Measurement

Nephelometry is based on the turbidity measurement of a solution in which an antibody–antigen complex is formed by mixing an antigen solution with an antibody. Within a defined analyte range and under defined conditions, this immune complex induces concentration-dependent light scattering. Due to its operation conditions, this immunological method qualifies as a fast and handy screening method for identifying A1PI-deficient patients. We here applied the nephelometric clinical test available for the ProSpec BN nephelometer (Siemens, Vienna, Austria). The Siemens test contains all the buffers required for the assay (N diluent REF OUMT65 and N reaction buffer REF OUMS65), the calibrated protein standard (N protein standard REF OQIM13SL), an assay control (REF OQIO13), and the N antiserum to human A1PI (REF OSAZ09). The six-point calibration curve ranges from 0.009 to 0.298 mg/mL. Plasma samples were measured using the dilution 1/20. A detailed method description and assay performance data are given in Appendix A.1.

### 4.4. A1PI Protein Measurement with the ELISA

An in-house developed and validated A1PI ELISA, which uses commercially available polyclonal antibodies, was applied. Coating buffer 1 (0.1 M NaHCO_3_, 0.1 M Na_2_CO_3_, pH 9.5), washing buffer (PBST, phosphate-buffered saline [PBS] with 0.05% Tween 20), dilution buffer 1 (DB1, 0.1% non-fat dry milk, 2 mM benzamidine), and a stopping solution (1.5 M H_2_SO_4_) were used. Maxisorp F96 flat-bottom plates (Nunc, VWR; Vienna, Austria) were coated with rabbit anti-human AAT IgG A0012 (DakoCytomation, Glostrup, Denmark), diluted to 10 µg/mL in coating buffer at 4 °C overnight. Bound A1PI was detected with sheep anti-human AAT IgG peroxidase (The Binding Site PP034; Birmingham, UK) and the peroxidase substrate SureBlue. Test samples and the assay standard (ERM DA 470 [59], 1.12 mg A1PI/mL) were diluted in DB1 and incubated in a serial dilution series with the plate at room temperature (RT) using the single incubation multilayer immune technique SIMIT [60]. The five-point assay calibration curve covered an A1PI concentration range from 28 to 1.8 ng/mL. Samples were measured in a dilution series containing six duplicate 1 + 1 dilutions. See Appendix A.2. for ELISA details and the assay’s performance data.

### 4.5. Functional A1PI Activity Measurement

Functional A1PI activity was measured with an in-house developed and validated elastase complex formation immunoassay assay (ECFISA) [61]. This assay relies on the complex formation between plate-bound porcine elastase and active A1PI. The quantity of complex formed is determined by detecting elastase-complexed and plate-bound A1PI using an anti-A1PI antibody, rather than measuring the activity of residual, non-inhibited elastase as in traditional chromogenic or fluorogenic assays. This unique detection method increases the assay’s sensitivity by a factor of approximately 1000. The assay’s selectivity is determined by the specificity of the elastase-A1PI complex formation, which remains unchanged even after elastase binds to a solid support. Additionally, it relies on the specificity of the anti-A1PI antibody used. Inactive A1PI, obtained through heat treatment, oxidation, or incubation with elastase, did not produce signals in the assay, thus confirming the assay’s specificity for measuring active A1PI.

The buffers used included ECFISA coating buffer (PBS, phosphate-buffered saline), washing buffer (PBST, PBS with 0.05% Tween 20), ECFISA dilution buffer 2 (DB2, 1% BSA in PBST), and stopping solution (1.5 sulfuric acid). Maxisorp F96 flat-bottom plates were coated with porcine elastase (Sigma, Vienna, Austria), diluted to 20 μg/mL with PBS at 4 °C overnight. A serial dilution series of the standard, a secondary in-house preparation calibrated against the WHO 1st international standard for alpha_1_-antitrypsin [62], and the samples were loaded to the wells and, after a PBST washing step, incubated with sheep anti-human AAT peroxidase (The Binding Site, UK, Birmingham). Bound peroxidase activity was next determined with SureBlue and sulfuric acid as the stopping solution. The assay calibration covered an A1PI activity range from 6 to 192 ng/mL. Refer to Appendix A.3. for further details on the ECFISA and data on the assay performance.

### 4.6. Urea Measurement

In clinical studies involving BAL samples, urea concentrations are measured in plasma and BAL samples to determine the volume of the original epithelial lining fluid diluted through the lavage procedure. We used the test combination Harnstoff (UV-Test; Boehringer Mannheim r-biopharm AG, Darmstadt, Germany). In this test, urea is hydrolyzed by urease to produce CO_2_ and NH_3_. The latter combines with 2-oxoglutarate and reduced nicotinamide adenine dinucleotide (NADH) in the presence of glutamate dehydrogenase to yield glutamate and NAD. The decrease in absorbance due to the decrease of NADH concentration over time is proportional to the urea concentration. The urea test is described in Appendix A.4. together with data on the assay performance.

## Data Availability

Data is contained in the paper.

## Abbreviations

The following abbreviations are used in this manuscript:

A1PI	α_1_-proteinase inhibitor
AAT	α_1_-antitrypsin
AATD	Alpha_1_-antitrypsin deficiency
BAL	Bronchoalveolar lavage
DB	Dilution buffer
ECFISA	Elastase complex formation immunosorbent assay
ELISA	Enzyme-linked immunosorbent assay
NADH	Reduced nicotinamide adenine dinucleotide
OD	Optical density
PBS	Phosphate-buffered saline
PBST	Washing buffer
RCL	Reactive center loop
RSD	Relative standard deviation
RT	Room temperature
SD	Standard deviation
Serpin	Serine protease inhibitor

## Appendix A

### Appendix A.1. Description of the Nephelometric A1PI Measurement

The nephelometer BN ProSpec (Siemens; Vienna, Austria) was used to measure human A1PI protein in human citrated plasma samples. The reference standard and the reagents provided with the instrument were used. The nephelometer runs the following procedure: A reference curve with the N protein standard SL (Siemens, Vienna, Austria) is created per test unit. This standard, with a defined, lot-specific A1PI concentration, is provided ready-to-use and stored at 4 °C. The calibration curve obtained comprises the six dilutions 1/5, 1/10, 1/20, 1/40, 1/80, and 1/160 (0.298 to 0.009 mg A1PI/mL). The calibration curve is automatically accepted if the mean deviation of the back-fitted concentrations of the calibration curve standards is ≤5%. In addition, the N/T protein control SL/M (Siemens, Vienna, Austria) is measured twice in independent dilutions. This control sample is also provided ready-to-use and is stored at 4 °C. The A1PI concentration determined must be within ± 15% of its labeled concentration for acceptance. In addition, the coagulation reference plasma pool standard 1R01 (Technoclone, Vienna, Austria) was established as an external assay control. This allowed us to run a control chart and check the inter-run assay precision independently of Siemens’ reagents. The additional control sample was measured at least once per test unit in a 1/20-dilution using the N diluent provided by Siemens. For the sample analysis, the nephelometer automatically starts with a 1/20 dilution. If a sample shows an A1PI concentration lower than the working range defined by the instrument, the sample is automatically re-analyzed using a 1/5 dilution. Table A1 summarizes assay performance data for the nephelometric A1PI protein measurement.

**Table A1:** Assay performance data for the nephelometric A1PI protein measurement.

Parameter	A1PI Level [mg/mL]	Number of A1PI Levels	Mean [Min–Max]
Accuracy [%recovery]	0.05–2.0	6	103.2% [100.0–107.5%]
Intra-run precision [RSD, n = 6]	0.05–2.0	6	0.8% [0.0–1.8%]
Inter-run precision [RSD, n = 6]	0.05–20	8	4.3% [3.4–6.5%]
Linearity	0.05–2.0	6	R^2^ = 1.0000
3× freezing-thawing [%recovery of fresh sample]	0.05; 0.5; 1.0	3	102.4% [98.8–105.7%]
48 h RT stability [%recovery of fresh sample]	0.05; 0.5; 1.0	3	96.6% [93.8–98.8%]

Remarks: Accuracy was determined by repeated measurement (n = 6) of six A1PI samples with defined A1PI concentrations ranging from 0.05 to 2 mg/mL. Intra- and inter-run precision determination relied on the repeated measurement (n = 6), carried out in one or six independent test units. Linearity was assessed by linear correlation analysis between expected and found A1PI concentrations ranging from 0.05 to 2 mg/mL. The stability of samples with A1PI concentrations of 0.05, 0.5, and 1 mg/mL was evaluated by subjecting them to three repeated freeze–thaw cycles and leaving them on the bench for 48 h at room temperature. RT stands for room temperature.

### Appendix A.2. Description of the A1PI Protein Measurement with the ELISA

The validated A1PI ELISA developed in-house uses these materials: 96-well Maxisorp F96 flat-bottom plates (Nunc, VWR; Vienna, Austria), polypropylene tubes (Greiner, Vienna, Austria), microtubes and disposable pipette tips (Bio-Rad, Vienna, Austria), and parafilm and reagent reservoirs (Costar, Vienna, Austria). The following chemicals were from VWR (Vienna, Austria): Na_2_CO_3_, NaHCO_3_, KCl, NaCl, KH_2_PO_4_, Na_2_HPO_4_ × 2 H_2_O, sulfuric acid (95–97%), water (HPLC grade), and HCl (25%). Non-fat dry milk was obtained from Maresi (Vienna, Austria), benzamidine hydrochloride monohydrate from Sigma (Vienna, Austria), Tween 20 (EIA grade) from Bio-Rad (Vienna, Austria), Patentblau V from Chroma-Waldeck (Münster, Germany), and the tetramethylbenzidine substrate SureBlue from KPL (Medac, Hamburg, Germany). The following buffers were used:

- Coating buffer: 0.1 M NaHCO_3_, 0.1 M Na_2_CO_3_; dissolved in HPLC-grade water; pH 9.5 with HCl (25%).
- Washing buffer (PBST): phosphate-buffered saline (PBS), 0.8% NaCl, 0.02% KCl, 0.02% KH_2_PO_4_, 0.126% Na_2_HPO_4_ × 2 H_2_O with 0.05% Tween 20; pH 7.0–7.4.
- Blocking/dilution buffer (DB): 0.1% non-fat dry milk, 2 mM benzamidine in PBST.
- Stopping solution: 1.5 M sulfuric acid.

The following biological materials were used:

- Rabbit anti-human α_1_-antitrypsin IgG A0012 (DakoCytomation, Glostrup, Denmark) as the capturing antibody.
- Sheep anti-human α_1_-antitrypsin IgG peroxidase PP034 (The Binding Site, Birmingham, UK) as the detection antibody.
- Human serum calibrator ERM DA 470, 1.12 mg A1PI/mL, as the assay calibrator.
- Human reference plasma 1A51 (Takeda, Vienna, Austria) as the control preparation.

The capturing antibody (rabbit anti-human α_1_-antitrypsin IgG) was diluted to 10 μg/mL in coating buffer, and 100 µL/well of this coating solution was incubated with a 96-well microplate at 4 °C overnight. The plate was then washed with PBST and incubated with 200 μL DB/well at 37 °C for 1 h to achieve blocking of the wells. Then, the serial dilution series of samples/calibrator/assay control was loaded into the wells (100 μL/well) and incubated at RT for 15 min. Subsequently, the detection antibody (sheep anti-AAT IgG peroxidase), diluted in DB, was added (100 μL/well), and the plate was incubated at RT for 1 h. A washing step terminated this incubation. Bound peroxidase was measured with SureBlue (100 μL/well) using 1.5 M sulfuric acid (100 μL/well) as a stopping solution. The plate was measured at 450 nm using a reference wavelength of 620 nm. Each plate underwent assay calibration. A linear regression model correlated the logarithms of the known A1PI concentrations of the assay calibrators and the logarithms of the blank-corrected optical densities (ODs) measured. The assay calibration curve consisted of a dilution series with five serial 1 + 1 dilutions of the assay calibrator ERM DA 470, ranging from 1/40,000 to 1/640,000, each measured in duplicate. Consequently, the calibration curve encompasses an A1PI concentration range from 28 to 1.8 ng/mL. Samples were analyzed in a dilution series with six duplicate 1 + 1 dilutions. The sample adsorptions must fall within the OD range specified by the calibration curve for further evaluation. Table A2 summarizes assay performance data for the A1PI protein measurement with the ELISA at low A1PI concentrations in samples with low total protein concentrations.

**Table A2:** Assay performance data for the A1PI protein measurement with the ELISA at low A1PI concentrations and low total protein concentrations.

Parameter	A1PI Level [µg/mL]	Number of A1PI Levels	Mean [Min–Max]
Accuracy [%recovery]	0.2–10	6	94.7% [88.9–99.2%]
Intra-run precision [RSD, n = 6]	0.2; 10	2	4.3% [3.3%; 5.2%]
Inter-run precision [RSD, n = 6]	0.2–10	6	7.0% [4.4–10.6%]
Linearity	0.2–10	6	R^2^ = 0.9993
3× freezing-thawing [%recovery of unfrozen]	0.2; 10	2	90.3% [85.7%; 94.9%]
48 h RT stability [%recovery of fresh]	0.2; 10	2	98.6% [97.1%; 100.0%]

Remarks: All validation samples had low total protein concentrations. Accuracy was determined by repeated measurement (n = 6) of six A1PI samples with defined A1PI concentrations ranging from 0.2 to 10 µg/mL. Intra- and inter-run precision determination relied on the repeated measurement (n = 6) carried out in one or six independent test units, respectively. Linearity was assessed by linear correlation analysis between expected and found A1PI concentrations ranging from 0.2 to 10 µg/mL. The stability of A1PI samples with concentrations of 0.2 and 10 µg/mL was evaluated by subjecting them to three repeated freeze–thaw cycles and leaving them on the bench for 48 h at room temperature. RT stands for room temperature.

Table A3 summarizes assay performance data for the A1PI protein measurement with the ELISA at normal plasma A1PI concentrations.

**Table A3:** Assay performance data for the A1PI protein measurement with the ELISA at normal plasma total protein concentrations.

Parameter	A1PI Level [mg/mL]	Number of A1PI Levels	Mean [Min–Max]
Accuracy [%recovery]	0.01–1.00	6	96.9% [94.0–103.7]
Inter-run precision [RSD, n = 6]	0.01–2.00	7	8.1% [6.2–9.4%]
Linearity	0.01–2.00	7	R^2^ = 0.9990

Remarks: All validation samples had a total protein concentration of about 50 mg/mL and were obtained by diluting a citrated human plasma pool with a human albumin solution. Accuracy was determined by repeated measurement (n = 6) of six A1PI samples with defined A1PI concentrations ranging from 0.01 to 1 mg/mL. Inter-run precision determination relied on the repeated measurement (n = 6) carried out in six independent test units. Linearity was assessed by linear correlation analysis between expected and found A1PI concentrations ranging from 0.01 to 2 mg/mL.

### Appendix A.3. Description of the A1PI ECFISA Measurement

The functional A1PI determination method uses solid phase-bound porcine elastase for measuring the anti-elastase inhibition activity of A1PI. The amounts of elastase-A1PI complex formed are directly measured by detecting elastase-complexed, plate-bound A1PI with an anti-A1PI antibody instead of measuring non-bound elastase activity as done in a conventional chromogenic assay. This mode of detection increases the assay’s sensitivity by a factor of approximately 1000.

The following buffers are required:

- ECFISA coating buffer (PBS): 8.0 g/L NaCl, 0.2 g/L KCl, 0.2 g/L KH_2_PO_4_, 1.26 g/L Na_2_HPO_4_ × 2 H_2_O. Salts are dissolved in 1 L HPLC water, pH checked (target pH 7.2 ± 0.2), and 0.2 µm filtrated with Nalgene filter unit (Sigma). The buffer can be stored at 4 °C for two weeks.
- Washing buffer (PBST): PBS with 0.05% (*v*/*v*) Tween 20. This washing buffer can be stored at RT for one week.
- ECFISA dilution buffer (DB): 5 g BSA (Sigma A0281) are dissolved in 500 mL PBST. The DB used for the dilution of samples contains 0.025% (g/v) Patentblau V, added from a 2.5% aqueous stock solution. Dilution buffer has to be prepared freshly before use.
- Peroxidase substrate SureBlue—ready to use.
- Stopping solution—3 N sulfuric acid.

Test sequence:

- Coating: Porcine elastase (Sigma, E7885) is dissolved in water (5 mg/mL) and kept frozen at −20 °C in 50 µL aliquots for up to 12 months. Immediately for the plate coating, an aliquot is thawed and diluted 1/250 with coating buffer. Nunc Maxisorp F96 plates are incubated with 100 µL/well coating solution at 4 °C overnight.
- Washing: Coating is terminated by a washing step with washing buffer done either manually or with a 96-well plate washer (Bio-Tek ELx-405). The washing is done three times; the emptied plate is then further processed.
- Blocking of wells: 200 µL/well ECFISA DB are added to the emptied wells using an 8-channel pipette or a dispenser (Multidrop 384 Dispenser). The plate is then incubated at 37 °C for 60 min. Blocking is terminated by a single washing step. The emptied plate is then further processed.
- Standard and sample dilution, loading and incubation: Each well is filled with 100 µL/well DB ECFISA. Colored DB ECFISA is used for the dilution of the standard and samples. The in-house assay standard with 19.2 mg A1PI/mL is diluted 1/50,000; samples are diluted to obtain AAT concentrations of about 400 ng/mL. A serial 1 + 1 dilution series comprising six dilutions is then prepared directly on the plate by mixing 100 µL of the colored sample dilution with the dilution buffer in the well. Two independent dilution series are prepared. The samples loaded to row B are measured in only five dilutions, as the positions B11 and B12 serve as blanks and contain DB only. Fading of the color with progressing dilution from the left to right side of the plate reflects the serial dilution series prepared. Each plate contains a dilution series for the assay standard, the assay control, and six samples. The dilutions (100 µL/well) are then incubated at RT for 60 min. Sample incubation is terminated by three washing steps, followed by a further three washing steps after the plate has been turned 180°. The emptied plate is then further processed.
- Incubation with anti-AAT peroxidase: Anti-human α_1_-antitrypsin peroxidase (TBS PP034) is diluted 1/1000 with DB. We added 100 µL/well and incubated at RT for 60 min. Incubation is terminated by a washing step, essentially carried out as described for the termination of the sample incubation.
- Color reaction: 100 µL/well SureBlue is added to the wells. The plate is incubated at RT for 15 min (protected from direct sunlight), before 100 µL/well stopping solution (3 N sulfuric acid) is added. Both additions can be done either manually or using the dispenser.
- Plate measurement: The plate is measured within 60 min with an ELISA reader (Bio-Tek EL-808) at 450 nm using a reference wavelength of 620 nm.
- Data evaluation: The calibration curve, ranging from 6 to 192 ng active A1PI/mL, is obtained as a linear regression curve calculated for the blank-corrected mean ODs of the duplicates and the AAT concentrations of the six assay standards. For the sample evaluation, only ODs within the range defined by the calibration curve are considered. The concentrations obtained for the individual samples’ dilutions are multiplied with the dilution and averaged to yield the final results.

Table A4 summarizes assay performance data for the functional A1PI measurement with the ECFISA at low A1PI concentrations in samples with low total protein concentrations.

**Table A4:** Assay performance data for the functional A1PI measurement with the ECFISA at low A1PI concentrations and low total protein concentrations.

Parameter	A1PI Level [µg/mL]	Number of A1PI Levels	Mean [Min–Max]
Accuracy [%recovery]	0.2–10	6	97.6% [85.0–107.4%]
Intra-run precision [RSD, n = 6]	0.2; 10	2	1.9% [1.4%; 2.4%]
Inter-run precision [RSD, n = 6]	0.2–10	6	4.5% [3.2–8.4%]
Linearity	0.2–10	6	R^2^ = 0.9998
3× freezing-thawing [%recovery of unfrozen]	0.2; 10	2	99.6% [99.1%; 100.0%]
4 h RT stability [%recovery of fresh]	0.2; 10	2	99.1% [98.1%; 100.0%]

Remarks: All validation samples had low total protein concentrations. Accuracy was determined by repeated measurement (n = 6) of six A1PI samples with defined A1PI concentrations ranging from 0.2 to 10 µg/mL. Intra- and inter-run precision determination relied on the repeated measurement (n = 6), carried out in one or six independent test units, respectively. Linearity was assessed by linear correlation analysis between expected and found A1PI concentrations ranging from 0.2 to 10 µg/mL. The stability of A1PI samples with concentrations of 0.2 and 10 µg/mL was evaluated by subjecting them to three repeated freeze–thaw cycles and leaving them on the bench for 4 h at room temperature. RT stands for room temperature.

Table A5 summarizes assay performance data for the functional A1PI measurement with the ECFISA at normal plasma total protein concentrations.

**Table A5:** Assay performance data for the functional A1PI measurement with the ECFISA at normal plasma total protein concentrations.

Parameter	A1PI Level [mg/mL]	Number of A1PI Levels	Mean [Min–Max]
Accuracy [%recovery]	0.01–3	7	101.8% [94.0–106.0%]
Intra-run precision [RSD, n = 6]	0.01–3	7	3.0% [2.0–4.2%]
Inter-run precision [RSD, n = 6]	0.01–20	9	6.3% [4.5–8.2%]
Linearity	0.01–3	7	R^2^ = 0.9993
3× freezing-thawing [%recovery of unfrozen]	0.01; 1	2	103.7% [104.5%; 102.9%]
4 h RT stability [%recovery of fresh]	0.01; 1	2	110.2% [106.6%; 113.8%]

Remarks: All validation samples had a total protein concentration of about 50 mg/mL and were obtained by diluting a citrated human plasma pool with a human albumin solution. Accuracy was determined by repeated measurement (n = 6) of seven A1PI samples with defined A1PI concentrations ranging from 0.01 to 3 mg/mL. Intra- and inter-run precision determination relied on the repeated measurement (n = 6), carried out in one or six independent test units, respectively. Linearity was assessed by linear correlation analysis between expected and found A1PI concentrations ranging from 0.01 to 3 mg/mL. The stability of A1PI samples with concentrations of 0.01 and 1 mg/mL was evaluated by subjecting them to three repeated freeze–thaw cycles and leaving them on the bench for 4 h at room temperature. RT stands for room temperature.

### Appendix A.4. Description of the Urea Measurement

The test combination Harnstoff (UV-Test; 10542946035), supplied by Boehringer/r-biopharm, was used according to the manufacturer’s instructions. In this test, urea is hydrolyzed in the presence of urease to produce CO_2_ and ammonia. By the action of glutamate dehydrogenase and in the presence of reduced nicotinamide adenine dinucleotide (NADH), the ammonia generated converts 2-oxoglutarate to L-glutamate, whereby NADH is consumed. The amount of NADH consumed during the reaction is equivalent to the amount of ammonia or half the amount of urea. NADH is measured and can be determined based on its absorption at 334, 340, or 365 nm.

The kit contains the following required reagents:

Vial 1: Solution of 2-oxoglutarate in triethanolamine buffer, pH about 8.0

Vial 2: NADH, 50 tablets

Vial 3: Urease solution

Vial 4: Solution of glutamate dehydrogenase.

One NADH tablet is dissolved in 1 mL of the solution of vial 1 to yield the reagent 1. When required, samples are diluted with aq. dest. 1 mL of the sample or control, pipetted in a single-use cuvette, and 0.5 mL of reagent 1 is added, followed by the addition of 10 μL urease solution (vial 3). The cuvette is then covered with parafilm and mixed through inversion. After incubation at RT for 5 min, the optical density (OD) is measured at 340 nm against a water blank to yield E1. Then, 10 μL of glutamate dehydrogenase solution (vial 4) are added. The cuvette is again covered with parafilm and mixed. The OD at 340 nm is measured after 20 min and yields E2. The blank-corrected decrease in OD (ΔE) is then used to calculate the sample’s urea concentration. Using an NADH extinction coefficient of 6.3 at 340 nm, the urea concentration is obtained in µg/mL according to the formula C_urea_ = 7.2453 × ΔE.

Table A6 summarizes assay performance data obtained for the urea measurement in a matrix with low protein content and in human citrated plasma samples.

**Table A6:** Assay performance data for the urea measurement.

Parameter	Urea Level [µg/mL]	Number of Urea Levels	Mean [Min–Max]
Accuracy [%recovery]	0.5–450	7	102.3% [97.5–110.7%]
Intra-run precision [RSD, n = 6]	0.5; 100; 300	3	7.6% [0.7–11.5%]
Inter-run precision [RSD, n = 6]	0.5–450	8	4.8% [0.6–9.1%]
Linearity—low protein	0.5–100	5	R^2^ = 0.9999
Linearity—plasma	150–450	3	R^2^ = 1.0000
Linearity—all samples	0.5–450	8	R^2^ = 1.0000
3× freezing-thawing [%recovery of unfrozen]	0.5; 100; 300	3	98.7% [96.2–101.9%]
4 h RT stability [%recovery of fresh]	0.5; 100; 300	3	98.5% [95.8–101.8%]

Remarks: Accuracy was determined by repeated measurement (n = 6) of seven samples with defined urea concentrations ranging from 0.5 to 450 µg/mL. Five of them had low protein content; the remaining two had the matrix of human citrated plasma. Intra- and inter-run precision determination relied on the repeated measurement (n = 6) carried out in one or six independent test units, respectively. Linearity was assessed by linear correlation analysis between expected and found urea concentrations. The stability of urea samples with concentrations of 0.5, 100, and 300 µg/mL was evaluated by subjecting them to three repeated freeze–thaw cycles and leaving them on the bench for 4 h at room temperature. RT stands for room temperature.

### Appendix A.5. Stability of A1PI Protein in Citrated Plasma as Measured with the Nephelometric Method

**Table A7:** Stability of A1PI in citrated plasma as measured with the nephelometric method.

**Months**		**Low**		**Normal**		**High**	
		**mg/mL**	**%Initial**	**mg/mL**	**%Initial**	**mg/mL**	**%Initial**
Storage at −20 °C	0	0.31	100.0	1.18	100.0	2.86	100.0
	3	0.31	100.0	1.29	109.3	3.11	108.7
	6	0.31	100.0	1.26	106.8	3.02	105.6
	9	0.32	103.2	1.34	113.6	3.26	114.0
	12	0.34	109.7	1.35	114.4	3.31	115.7
	15	0.31	100.0	1.28	108.5	3.22	112.6
	18	0.32	103.2	1.27	107.6	3.01	105.2
	24	0.33	106.5	1.22	103.4	3.14	109.8
Storage at −60 °C	0	0.31	100.0	1.18	100.0	2.86	100.0
	3	0.31	100.0	1.30	110.2	3.12	109.1
	6	0.31	100.0	1.22	103.4	2.95	103.1
	12	0.32	103.2	1.28	108.5	3.16	110.5
	15	0.31	100.0	1.22	103.4	3.09	108.0
	18	0.32	103.2	1.22	103.4	3.04	106.3
	24	0.31	100.0	1.20	101.7	3.04	106.3

### Appendix A.6. Stability of A1PI Protein in Citrated Plasma as Measured with the ELISA

**Table A8:** Stability of A1PI in citrated plasma as measured with the ELISA.

Months		Low		Normal		High	
		mg/mL	%Initial	mg/mL	%Initial	mg/mL	%Initial
Storage at −20 °C	0	0.31	100.0	1.28	100.0	3.54	100.0
	3	0.28	90.3	1.29	100.9	3.34	94.4
	6	0.30	96.8	1.16	90.7	3.44	97.2
	9	0.35	112.9	1.39	108.7	3.92	110.7
	12	0.34	109.7	1.41	110.2	4.11	116.1
	15	0.29	93.5	1.21	94.6	3.20	90.4
	18	0.29	93.5	1.32	103.2	3.28	92.7
	24	0.31	100.0	1.22	95.4	3.00	84.7
Storage at −60 °C	0	0.31	100.0	1.28	100.0	3.54	100.0
	3	0.28	90.3	1.21	94.6	3.28	92.7
	6	0.30	95.7	1.27	99.3	3.60	101.8
	12	0.30	95.7	1.26	98.8	3.62	102.2
	15	0.29	94.6	1.22	95.4	3.39	95.9
	18	0.32	102.2	1.33	104.0	3.40	96.0
	24	0.32	104.3	1.26	98.5	3.53	99.6

### Appendix A.7. Stability of Functional A1PI Activity in Citrated Plasma

**Table A9:** Stability of functional A1PI activity in citrated plasma.

Months		Low		Normal		High	
		µg/mL	%Initial	µg/mL	%Initial	µg/mL	%Initial
Storage at −20 °C	0	241	100.0	1027	100.0	2889	100.0
	3	255	105.9	865	84.2	2901	100.4
	6	261	108.3	1167	113.6	3023	104.7
	9	222	92.2	952	92.7	2385	82.6
	12	222	92.2	909	88.5	2483	86.0
	15	282	117.1	1068	104.0	2674	92.6
	18	269	111.7	967	94.2	2733	94.6
	24	259	107.5	857	83.4	2439	84.4
Storage at −60 °C	0	241	100.0	1027	100.0	2889	100.0
	3	271	112.5	1042	101.5	2929	101.4
	6	280	116.2	1148	111.8	3145	108.9
	12	245	101.7	999	97.3	2597	89.9
	15	287	119.1	1187	115.6	2882	99.8
	18	276	114.6	1025	99.8	2970	102.8
	24	251	104.2	1045	101.7	2824	97.8

### Appendix A.8. Stability of A1PI Protein in BAL Mock Samples

**Table A10:** Stability of A1PI protein in BAL mock samples.

Months		1 µg/mL		10 µg/mL	
		µg/mL	%Initial	µg/mL	%Initial
Storage at −20 °C	0	0.98	100.0	11.5	100.0
	3	1.00	101.7	12.6	109.3
	6	1.10	111.9	12.8	111.0
	9	0.96	98.0	11.9	103.5
	12	1.07	108.8	12.6	109.9
	15	0.90	92.2	12.6	109.3
	18	0.99	101.0	12.4	107.5
Storage at −60 °C	0	0.98	100.0	11.5	100.0
	3	1.03	105.1	12.4	108.1
	6	1.04	105.8	12.2	105.8
	9	0.97	99.3	13.0	113.0
	12	0.91	93.2	11.6	100.6
	15	1.01	103.4	13.2	114.5
	18	1.00	102.4	12.8	111.0

### Appendix A.9. Stability of Functional A1PI Activity in BAL Mock Samples

**Table A11:** Stability of functional A1PI activity in BAL mock samples.

Months		1 µg/mL		10 µg/mL	
		µg/mL	%Initial	µg/mL	%Initial
Storage at −20 °C	0	0.99	100.0	12.8	100.0
	3	0.90	91.2	11.7	91.3
	6	0.92	92.9	11.5	89.6
	9	0.82	82.8	11.0	85.9
	12	0.91	92.3	11.4	89.3
	15	0.89	89.9	11.1	87.0
	18	0.74	74.7	11.2	87.8
Storage at −60 °C	0	0.99	100.0	12.8	100.0
	3	0.90	90.5	11.4	88.8
	6	0.94	95.3	11.2	87.5
	9	0.84	84.5	12.4	96.6
	12	0.82	82.5	10.6	83.1
	15	0.93	93.6	11.5	89.6
	18	0.72	73.1	9.45	73.9

### Appendix A.10. Stability of Urea in BAL Mock Samples and Citrated Human Plasma

**Table A12:** Stability of urea in BAL mock samples.

Months	0.5 µg/mL		5 µg/mL		100 µg/mL	
	µg/mL	%Initial	µg/mL	%Initial	µg/mL	%Initial
0	0.55	100.0	5.12	100.0	102	100.0
3	0.54	98.2	5.38	105.1	102	100.0
6	0.66	120.0	5.32	103.9	102	100.0
12	0.62	112.7	5.43	106.1	104	102.0
18	0.60	109.1	5.43	106.1	108	105.9

**Table A13:** Stability of urea in citrated human plasma.

Months	Low A1PI		Normal A1PI		High A1PI	
	µg/mL	%Initial	µg/mL	%Initial	µg/mL	%Initial
0	61	100.0	238	100.0	205	100.0
3	59	96.7	225	94.5	199	97.1
6	56	91.8	262	110.1	179	87.3
9	64	104.9	222	93.3	201	98.0
12	59	96.7	205	86.1	201	98.0
15	57	93.4	229	96.2	187	91.2
18	55	90.2	219	92.0	200	97.6
24	61	100.0	239	100.4	191	93.2
